# Metformin Suppresses Hypopharyngeal Cancer Growth by Epigenetically Silencing Long Non-coding RNA SNHG7 in FaDu Cells

**DOI:** 10.3389/fphar.2019.00143

**Published:** 2019-02-22

**Authors:** Ping Wu, Yaoyun Tang, Xing Fang, Chubo Xie, Junfeng Zeng, Wei Wang, Suping Zhao

**Affiliations:** Department of Otorhinolaryngology Head and Neck Surgery – Province Key Laboratory of Otolaryngology Critical Diseases, Xiangya Hospital of Central South University, Changsha, China

**Keywords:** hypopharyngeal cancer, metformin, long non-coding RNAs, DNA methylation, SNHG7

## Abstract

Local recurrence after therapy remains a challenging problem for hypopharyngeal cancer (HPC) due to the chemotherapy resistance. Metformin is associated with reduced cancer risk through promoting global DNA methylation in cancer cells by controlling *S*-adenosylhomocysteine (SAHH) activity. However, the mechanisms by which metformin inhibits HPC remain elusive. In this study, we aim to investigate the role of metformin in HPC and illustrate the mechanism by which metformin regulates long non-coding RNAs (lncRNAs) expression. CCK-8 and annexin-V/PI double staining were performed to analyze the cell viability and apoptosis. LncRNA microarray analysis, QPCR, methylation specific PCR, Western blot and RNA Immunoprecipitation were performed to analyze the molecular mechanism, Here, we report that metformin inhibits FaDu cell proliferation in time- and dose-dependent manner by suppressing lncRNA SNHG7. Further investigations revealed that SNHG7 interacted with SAHH and metformin decreased SNHG7 expression by activating SAHH activity. Increased SAHH activity resulted in upregulating DNMT1 expression, leading to hypermethylation of SNHG7 promotor. In addition, upregulation of SNHG7 was associated with advanced stage. The patients with high SNHG7 have lower overall survival than that of with low SNHG7. Interestingly, SNHG7 levels were higher in taxol resistant patients than in taxol sensitive patients. Metformin sensitizes FaDu cells to taxol and irradiation through decreasing SNHG7. In conclusion, our recent study demonstrates that metformin inhibits FaDu cell proliferation by decreasing SNHG7 expression via SAHH-mediated DNA methylation. These findings indicate that combined metformin with paclitaxel or irradiation would be a novel therapeutic strategy to overcome resistance and prevent recurrence in HPC.

## Introduction

In early-staged hypopharyngeal cancer (HPC), the overall and disease-specific survival rates after organ-preserving adjuvant chemoradiotherapy is satisfied (Chen et al., 2013). However, HPC still remains one of the worst prognose cancers with high mortality (Chan and Wei, 2013). Chemoradiotherapy is the first line treatment for advanced HPC (Bradley and Bradley, 2012). However, local recurrence remains an issue for HPC due to the chemotherapy resistance (Newman et al., 2015).

Metformin, a first-line drug for type 2 diabetes, is associated with reduced cancer risk. Increasing evidence has suggested that it is a potential anti-tumor drug (Pan et al., 2015). It was reported that metformin promoted global DNA methylation by decreasing *S*-adenosylhomocysteine (SAHH) (Cuyas et al., 2018). DNA methylation may induce dysregulated gene expression. Metformin treatment could reduce the levels of histone methyltransferase of H3 Lys9 (SUV39H1) to inhibit migration of prostate cancer cells (Yu et al., 2017). However, the mechanisms by which metformin inhibits HPC remain elusive.

Long non-coding RNAs (lncRNAs) play a vital role in human cancers by serving as either tumor oncogenes or tumor suppressor genes through regulating cell growth, apoptosis, migration, and invasion (Guo et al., 2017). Previous study has shown that lncRNAs were differentially expressed in patients with hypopharyngeal squamous cell carcinoma and radioresistant HPC cells (Zhou et al., 2015a, 2017). LncRNA PEG10 highly expresses in HPC tissues compared with para-carcinoma tissues, and was associated with advanced stage. Enhanced expression of lncRNA PEG10 increases cell growth and metastasis of FaDu cells (Zhao et al., 2017). LncRNA UCA1 functions as an oncogene in HPC cells (Qian et al., 2017). In addition, metformin exposure inhibits endometrial cancer cell proliferation and leads to hypermethylation of tumor-promoting pathway genes, partly by downregulating H19 via DNA methylation (Yan et al., 2015). Metformin enhanced the effectiveness of photon irradiation in glioblastoma cells. Cell toxicity was more pronounced in O6-methylguanine DNA methyltransferase (MGMT) promoter non-methylated glioblastoma cell (Adeberg et al., 2017). These findings suggest that metformin may sensitize HPC to chemotherapy by epigenetic pathway.

In present study, we aim to investigate the role of metformin in HPC and illustrate the mechanism by which metformin regulates lncRNAs expression. Here, we report that metformin inhibits FaDu cell proliferation in time- and dose-dependent manner by suppressing lncRNA SNHG7 through epigenetic mechanism. In addition, metformin sensitizes FaDu cell to taxol through decreasing SNHG7. Thus, our study reports a novel mechanism underlying metformin inhibits carcinogenesis and provides a potential novel diagnosis and treatment biomarker for HPC.

## Materials and Methods

### Cell Culture and Treatment

Hypopharyngeal cancer cell line, FaDu cells, was obtained from National Infrastructure of Cell Line Resource (Beijing, China). Cells were cultured in RP1640 medium supplemented with 10% fetal bovine serum under 5% CO_2_ and 37°C conditions.

The taxol-resistant FaDu cell line (FaDu/taxol) was established in our lab. The FaDu cells (1 × 10^5^/ml) were seeded and further cultured for 24 h. With ∼80% confluence, cells were treated with 5 nM taxol. The medium was changed with fresh completed medium every 3 days. Each dose was maintained for 2 weeks, and then was doubled. The final concentration was increased to 40 nM.

FaDu cells were exposed to metformin at a series of concentrations (0, 2, 4, 6, 8 mM) for 48 h or at 8 mM for various hours (0, 12, 24, 36, 48, 72).

Pre-treated cells with or without 8 mM metformin and lentivirus expressing SNHG7 for 24 h were inoculated onto 6-well plates. The cells were irradiated (0, 2, 4, and 6 Gy, 2 Gy/min) using a SIEMENS linear accelerator (SIEMENS Medical Systems, Germany). The cells were cultured for additional 24 h and then used for CCK-8 assay and apoptosis analysis.

### Cell Infection and Transfection

To generate lentivirus expressing SNHG7 (Lv-SNHG7), the lentiviral transduction particles for SNHG7 were constructed and purchased from Shanghai Genechem Co., Ltd. (Shanghai, China). To generate lentivirus expressing shDNMT1 (Lv-shDNMT1, target sequences: CCGGGACGACCCTGACCTCAAATATCTCGAGATATTTGAGGTCAGGGTCGTCTTTTTG), the lentiviral transduction particles for shDNMT1 were purchased from Merk (Shanghai, China). 2 × 10^5^ cells per well were seeded. With reached 80–90% confluence, the cells were infected with lentivirus [multiplicity of infection (MOI) = 50] supplemented with 5 mg/ml polybrene (Sigma-Aldrich, St. Louis, MO, United States) for 48 h.

### RNA Extraction and RT-qPCR

Total RNA was extracted using TRIzol reagent (Invitrogen). Total RNA was reversed transcript using Maxima First Strand cDNA Synthesis kit (Thermo Fisher Scientific, Inc.) according to the manufacturer’s protocol. Quantitative polymerase chain reaction (qPCR) was performed on an CFX96 Touch Deep Well Real-Time PCR Detection System (Bio-Rad, Hercules, CA, United States). Expression of SNHG7, DNMT1, GALNT1, Cyclin D1, FAIM2, p15, p16, MDR1, MRP7, LRP, BCRP, and TRAG3 was measured using UltraSYBR Mixture (CWBio, Wuhan, China). The thermocycling conditions as following: denaturation at 95.0°C for 3 min; 39 cycles of 95.0°C for 10 s and 60°C for 30 s. The comparative Ct method formula 2^-ΔΔCt^ was used to calculated relative gene expression. Expression of β-actin was used as an endogenous control. The primer sequences are shown in Table 1.

**Table 1:** Primers used for RT-qPCR.

Genes name	Sequences (5′–3′)
SNHG7	Forward: GTGTGTCCCTTGGTGGAGAG Reverse: TCCCAGATACCAGCGAAGGA
LINC01504	Forward: AGAGCGTGGCTTTAACGTCT Reverse: CCAGGGCCATCTGAGCATAC
LINC00189	Forward: GGGAACAACAGGTTGCCTTAG Reverse: GCATCCTTCCTGTGTCATCCA
DNMT1	Forward: AGGAGGGCTACCTGGCTAAA Reverse: CGTCTCCATCTTCGTCCTCG
DNMT2	Forward: GATTGAAGATTGGCCGGCAG Reverse: GCACATCCTCTGCAGTCTGT
DNMT3A	Forward: CGAGAGCAGAGGACGAGC Reverse: AGCAGACCTTTAGCCACGAC
DNMT3B	Forward: CCCCTCAAACCCATTCCCTC Reverse: GCATCCGTCATCTTTCAGCC
GALNT1	Forward: GGATTTGGCAGTGTGGAGGA Reverse: TTTCTGGCAGGGTGACGTTT
Cyclin D1	Forward: CTGATTGGACAGGCATGGGT Reverse: GTGCCTGGAAGTCAACGGTA
FAIM2	Forward: CAGGCAGGCTGCGGTTAC Reverse: GCATAGGATGCCCAGTACCA
p15	Forward: GGAGTTAATAGCACCTCCTCC Reverse: TTCAATCGGGGATGTCTGAGG
p16	Forward: AGGAAAAGCCCGGAGCTAAC Reverse: CATCATCATGACCTGGATCGC
MDR1	Forward: CCTGTGAAGAGTAGAACATGAAGA Reverse: CGAATGAGCTCAGGCTTCCT
MRP7	Forward: CGGCTAGGTCTTCCAACCTC Reverse: GGTGGCAAAGCAACTGGAAG
LRP	Forward: CTGCAGGCCAACACCATCA Reverse: GCCCAAAGGCTGTGTTGAAG
BCRP	Forward: CGCACAGAGCAAAGCCATTT Reverse: TTCCTTCCTGCCTGCTCTTG
TRAG3	Forward: AGACATAACTTCAGGTGACACA Reverse: GGGTTGTCTTGGGAACCTCT


### LncRNA Microarray Analysis

Total RNA was extracted from cells treated with metformin or vehicle by the RNeasy Mini Kit (Qiagen, GmBH, Hilden, Germany) according to the manufacturer’s instructions. Purified total RNA was quantified using the NanoDrop 2000 spectrophotometer. LncRNA expression profiles was conducted by Novogene Co., Ltd. (Beijing, China). Differentially expressed lncRNAs were identified through fold change.

### Western Blot

RIPA lysis buffer supplemented with a proteinase inhibitor cocktail (Boster, Wuhan, China) was used to extract protein. BCA Protein assay kit (Thermo Scientific, Waltham, MA, United States) was used to measure protein concentration. Protein were separated by 10% SDS/PAG and transferred to PVDF membrane. The membranes were incubated with following primary antibodies (SAHH antibody, diluted at 1:1000; DNMT1 antibody, diluted at 1:2000; DNMT2 antibody, diluted at 1:1000; DNMT3A antibody, diluted at 1:2000; DNMT3B antibody, diluted at 1:1000; p-AMPK antibody, diluted at 1:1000; AMPK antibody, diluted at 1:1000; GAPDH antibody, diluted at 1:3000; all the antibodies were purchased from Cell Signaling Technology). Membranes were then incubated with appropriate secondary antibodies. The bands were detected using a Bio-Rad (Hercules, CA, United States) imaging system.

### Proliferation Assay

#### Cell Counting Kit-8 (CCK-8) Assay

0.5 × 10^4^ cell per well were seeded in 96-well plate. The treated cells were cultured either 24 h, 48 h or 72 h. Ten microliters of CCK-8 reagents (Beyotime, Hangzhou, China) were added. Following 1-h culture, OD value (570 nm) was determined by a microplate reader.

#### BrdU Assay

The metformin treated cells were cultured with BrdU (final dilution, 1:4000, catalog no. 11647229001; Merck) for 24 h according to the manufacturer’s instructions. The experiment was done twice with triplicate samples.

### Annexin-V/PI Double Staining for Apoptosis Analysis

Cell apoptosis was determined by Annexin V apoptosis detection kit (Life technologies, Grand Island, NY, United States). FaDu cells were collected and resuspended in 500 μl binding buffer. The cells were incubated with 10 μl Annexin V-FITC and 10 μl propidium iodide for 15 min. Then, the cells were analyzed using flow cytometric analysis (BD Biosciences, San Jose, CA, United States).

### Tumor Xenograft in Nude Mice

Animal experiments were approved by the Ethical Committee for Animal Research of the Xiangya Hospital of Central South University. The FaDu cells were treated with metformin or combined with SNHG7 lentivirus infection. The 100 μl treated cells (1 × 10^6^ cells) were subcutaneously injected into left rib of nude mice (*N* = 5 per group, 2 months-old). The tumor sizes were recorded and calculated using the formula: 0.5 × *L* × *W*^2^, where *L* and *W* are the long and short diameter of the tumor.

### RNA Immunoprecipitation (RIP)

Magna RIP RNA-Binding Protein IP Kit (Millipore, Bedford, MA, United States) was used for RNA immunoprecipitation experiments. The cells were lysed in complete RIP lysis buffer for 30 min. The cell extract was incubated with the mix containing RIP buffer and magnetic beads conjugated with anti-SAHH antibody (Cell Signaling) overnight at 4°C. Normal mouse IgG (Millipore) was used as negative control. Purified RNAs in the precipitates were used to determine SNHG7 expression.

### Methylation Specific PCR (MSP)

Methylation status of SNHG7 promoter was measured by MSP. Qiagen FFPE DNA Kit (Qiagen, CA, United States) was used to extract genomic DNA. EZ DNA Methylation-Gold Kit (Zymo, Orange County, CA, United States) was used to modify genomic DNA with bisulfite according to the manufacturer’s instructions. Bisulfate-treated DNA was used for quantitative methylation-specific PCR (qMSP). The qPCR thermocycling conditions were the same as mentioned above.

**FIGURE 1:**
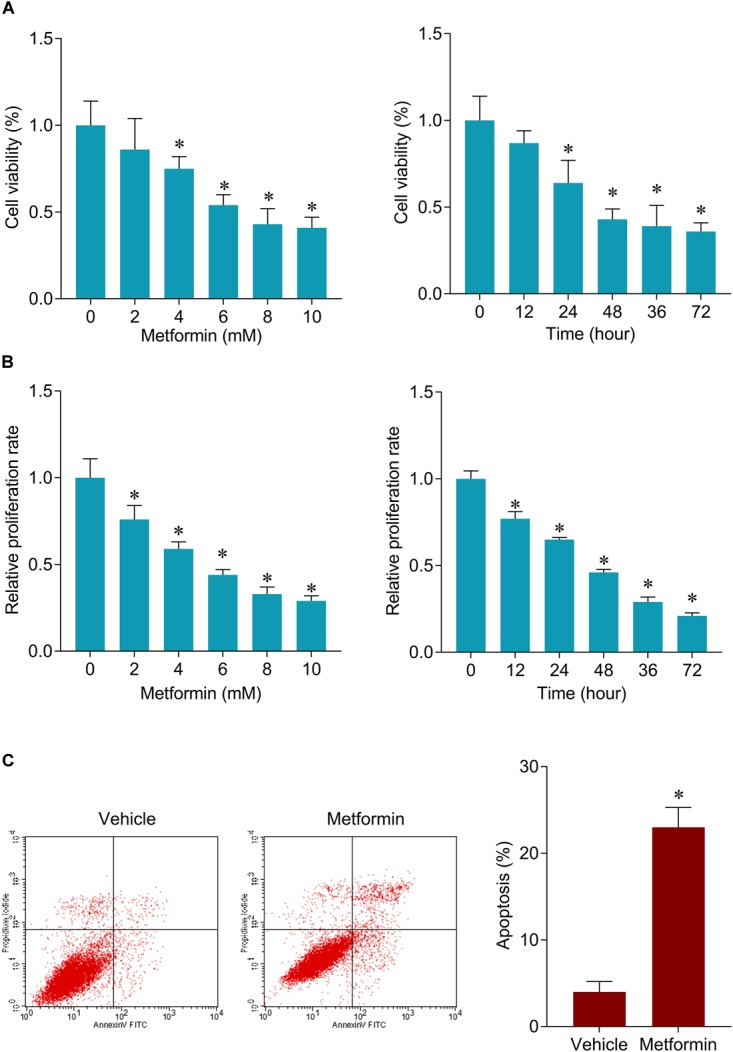
Metformin inhibits cell viability and induces apoptosis in FaDu cells. **(A)** CCK-8 assay was used to determine the cell viability after 48 h of a serial concentrations of metformin (left) treatment and after 8 mM metformin treatment for a serial of time points (right) in FaDu cells. **(B)** BrdU assay was used to determine the cell proliferation rate in FaDu cells. **(C)** Flow cytometry was used to measured cell apoptosis after 8 mM metformin treatment for 48 h in FaDu cells. ^∗^*P* < 0.05.

### SAHH Activity Assay

Human homocysteine (Hcy) ELISA Kit (cat no. MBS260128, Mybiosource, San Diego, CA, United States) was used to perform SAHH activity assay according to the manufacturer’s instructions. Briefly, FaDu cells were washed with PBS and lysed in 200 μl of lysis buffer. Following 15 min centrifugation at 15,000 × *g* at 4°C, SAHH activity was measured in 100 μl supernatant using a microplate reader.

### Tissue Samples

Seventy-three HPC tissues with clinical staging and survival information and the matched adjacent tissues were collected. The taxol sensitive patients were defined as had prolonged stable disease of more than 6 months or a partial response and complete response to chemotherapy containing taxol. The taxol resistant patients were defined as had stable disease less than 6 months after chemotherapy containing taxol in the first setting. Written informed consent was obtained from the participants of this study. This project was approved by the Ethics Committee of The Xiangya Hospital of Central South University.

**FIGURE 2:**
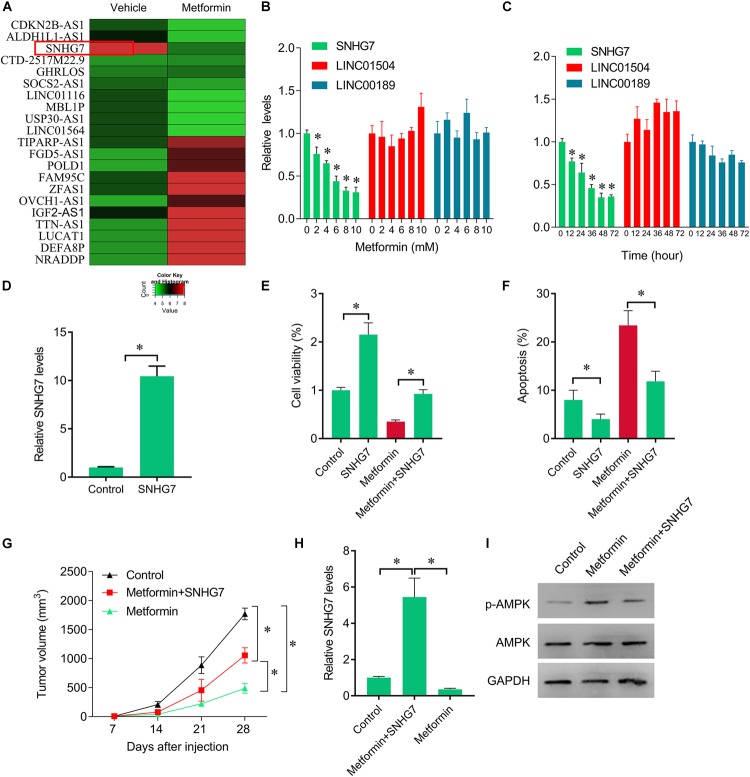
Rescue of SNHG7 reverse metformin-mediated inhibitory effects *in vitro* and *in vivo*. **(A)** The heatmap showed the differentially expression lncRNAs in metformin (8 mM for 48 h) treated FaDu cells. SNHG7 was significantly decreased by metformin compared with vehicle treated cells. **(B)** QPCR was performed to measure the SNHG7, LINC01504, and LINC00189 expression after 48 h of a serial concentrations of metformin treatment in FaDu cells. **(C)** QPCR was performed to measure the SNHG7, LINC01504, and LINC00189 expression after 8 mM metformin treatment for a serial of time points in FaDu cells. **(D)** QPCR was performed to measure the SNHG7 expression after SNHG7 lentivirus infection. **(E)** CCK-8 assay was used to determine the cell viability after metformin treatment (8 mM for 48 h) or combined with SNHG7 lentivirus. **(F)** Flow cytometry was used to measured cell apoptosis after metformin treatment (8 mM for 48 h) or combined with SNHG7 lentivirus. **(G)** FaDu cells treated with metformin or together with SNHG7 lentivirus were injected into nude mice. The cells untreated were used as negative control. Tumor volumes were calculated and shown. **(H)** QPCR was performed to measure the SNHG7 expression in tumor tissues from nude mice. **(I)** Western blot was performed to measure the p-AMPK and AMPK expression in tumor tissues from nude mice. ^∗^*P* < 0.05.

### Statistical Analysis

Statistical analysis was performed on GraphPad Prism software (GraphPad Software Inc., La Jolla, CA, United States). Values are expressed as means ± SEM. Student’s *t*-test was used to compare differences between two groups. Differences among multiple groups was evaluated by one-way ANOVA with Bonferroni test. Kaplan–Meier method with log-rank test was used to estimate the overall survival rate. *P*-value less than 0.05 was accepted as statistical significant.

## Results

### Metformin Inhibits FaDu Cells Growth Through Suppressing SNHG7

The inhibitory roles of metformin have been illustrated in several cancers. We investigated the role of metformin HPC. We found that metformin treatment significantly inhibited FaDu cell viability and proliferation in a dose- and time-dependent manner (Figure 1A,B). The apoptosis rate in FaDu cells treated with 8 mM metformin for 48 h was significantly increased compared with control cells (Figure 1C). We further investigated whether the inhibitory roles of metformin in HPC through lncRNAs. LncRNAs microarray was performed to screen the differentially expressed lncRNAs in metformin (8 mM for 48 h) treated cells. Among the hundreds differentially expressed lncRNAs (Figure 2A), of which 10 top-upregulated lncRNAs were shown in Table 2. We confirmed the expression of lncRNA SNHG7, LINC01504, and LINC00189 by qPCR in cells after metformin treatment, and found that metformin reduced SNHG7 expression, but not LINC01504 and LINC00189, in a dose- and time-dependent manner (Figure 2B,C). Thus, we focused on the lncRNA SNHG7, which has been shown to act as a tumor suppressor in lung cancer (She et al., 2016). We overexpressed the expression of SNHG7 in FaDu cells (Figure 2D) and observed that upregulation of SNHG7 significantly increased the cell viability and reduced the apoptosis rate mediated by metformin treatment compared with that of in cells treated with metformin alone (Figure 2E,F). The inhibitory effects were also confirmed *in vivo*. SNHG7 significantly increased tumor volume, which were suppressed by metformin treatment (Figure 2G). We also confirmed that SNHG7 was repressed by metformin and upregulated by lentivirus infection (Figure 2H), and SNHG7 upregulation inhibited metformin-activated AMPK signaling (Figure 2I). Thus, these results suggest that metformin inhibits FaDu cells growth through suppressing SNHG7.

**Table 2:** The detail information of the top 10 down-regulated lncRNAs.

Downregulated lncRNAs	Fold changes (metformin/vehicle)	*P*-value
SNHG7	-15.58	0.001204
LINC01504	-12.69	0.001304
LINC00189	-8.49	0.002849
SNHG4	-6.53	0.000937
LOC731157	-5.09	0.004459
LINC00884	-4.86	0.002347
MTERF4	-4.70	0.003798
TRAF3IP2-AS1	-4.50	0.013113
HIPK1-AS1	-3.70	0.001862
LOC729218	-3.23	0.001203


**FIGURE 3:**
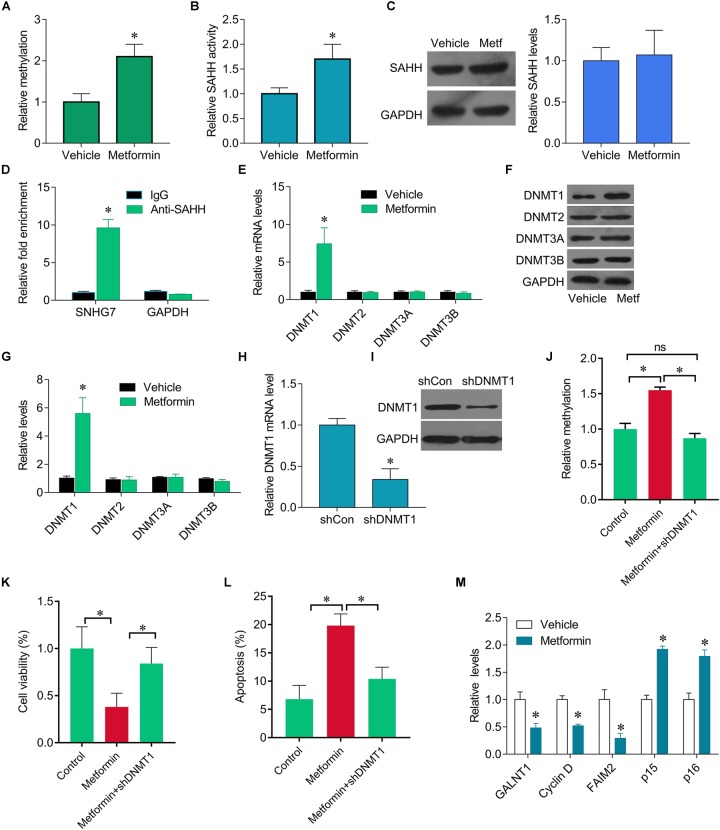
Metformin epigenetically regulates SNHG7. **(A)** qMSP test results indicated that methylation of SNHG7 promoter was higher in metformin treated FaDu cells than that of in vehicle treated cells. **(B)** FaDu cells were treated with or without metformin (metf) at a final concentration of 8 mM for 48 h. SAHH activity were assessed by SAHH activity assay. **(C)** SAHH protein levels were assessed by Western blot. **(D)** RNA immunoprecipitation (RIP) was performed. The levels of SNHG7 and GAPDH mRNA are presented as fold enrichment in anti-SAHH relative to IgG immunoprecipitants. **(E)** qPCR was performed to measure the expression of DNMT1, DNMT2, DNMT3a and DNMT3b after metformin treatment. **(F,G)** DNMT1, DNMT2, DNMT3a, and DNMT3b protein levels were assessed by Western blot in FaDu cells treated with or without metformin (metf) **(F)** and the quantification of bands **(G)**. **(H)** Confirmation of Dnmt1 knockdown by RT–qPCR. **(I)** Confirmation of Dnmt1 knockdown by Western blot analysis. **(J)** qMSP analysis of SNHG7 promoter methylation. **(K,L)** Cell viability **(K)** and cell apoptosis rate **(L)** were assessed by CCK8 and flow cytometry in FaDu cells treated metformin with or without shDNMT1. **(M)** RT-qPCR analysis for GALNT1, Cyclin D1, FAIM2, p15, p16 in FaDu cells after treated with or without metformin. ^∗^*P* < 0.05.

### Metformin Mediates Hypermethylation of SNHG7 Promoter

We further investigate how metformin control the expression of SNHG7. Previous studies have been shown metformin could epigenetically regulate genes expression. We found that SNHG7 promoter was hypermethylated in metformin treated cells (Figure 3A). Furthermore, we observed that metformin induced the activity of SAHH, but not altered its expression (Figure 3B,C). The RIP experiments demonstrated the interaction of SAHH and SNHG7 (Figure 3D). Meanwhile, metformin significantly upregulated DNMT1 expression but not DNMT2, DNMT3A, and DNMT3B at mRNA and protein levels (Figure 3E–G). Interestingly, we knocked down of DNMT1 in FaDu cells (Figure 3H,I). DNMT1 downregulation diminished hypermethylation of SNHG7 promoter mediated by metformin (Figure 3J). Moreover, DNMT1 downregulation reversed metformin-mediated inhibition of cell growth (Figure 3K,L). We also found that metformin treatment could control the downstream molecular expression, such as decreasing GALNT1, cyclin D1 and FAIM2, and increasing p15 and p16 (Figure 3M). These evidences indicate that metformin can epigenetically regulate SNHG7 expression in HPC cells.

### High SNHG7 Is Associated With Advanced Hypopharyngeal Cancer

**FIGURE 4:**
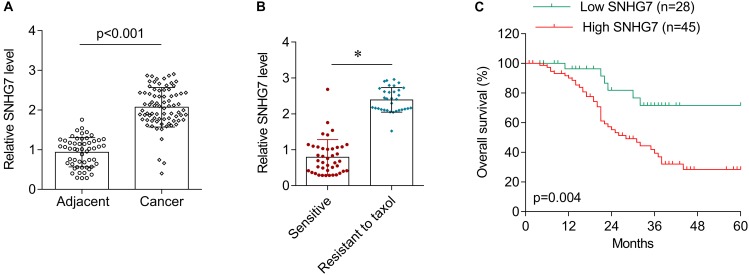
The expression of SNHG7 in hypopharyngeal cancer tissues. **(A)** RT-qPCR was used to determine the expression of SNHG7 in hypopharyngeal cancer tissues (*N* = 73) and matched adjacent control (*N* = 73). **(B)** The expression of SNHG7 in patients who sensitive (*N* = 38) or primary resistant (*N* = 33) to taxol. **(C)** Overall survival analysis in hypopharyngeal cancer patients with low or high SNHG7 expression. ^∗^*P* < 0.05.

SNHG7 expression was significantly increased in HPC tissues compared with adjacent control (Figure 4A). SNHG7 expression was higher in patients who sensitive to taxol than in patients who primary resistant to taxol (Figure 4B). The patients were divided into high SNHG7 and low SNHG7 groups according to the median of SNHG7 expression. High SNHG7 expression was associated with tumor size (*p* = 0.033), differentiation (*p* = 0.044), lymph node metastasis (*p* = 0.013), distant metastasis (*p* = 0.017) and TNM stage (*p* = 0.045), but not associated with age and gender (Table 3). Univariate analysis indicated that the SNHG7 level (*p* = 0.013) was significantly associated with patients’ prognosis (Table 4). Multivariate analysis revealed that SNHG7 (*p* = 0.024) was an independent prognosis factor for HPC patients (Table 5). In addition, the patients with low SNHG7 have longer overall survival time than the patients with high SNHG7 (Figure 4C).

**Table 3:** Association between SNHG7 levels and clinicopathological variables of patients with hypopharyngeal cancer.

	SNHG7		
		
Variable	Low expression (*n* = 28)	High expression (*n* = 45)	χ^2^ test *p*-value
**Age**			0.632
<60	13	18	
≥60	15	27	
**Gender**			0.625
Male	18	25	
Female	10	20	
**Tumor size**			0.033
<3 cm	18	17	
≥3 cm	10	28	
**Differentiation**			0.044
High	11	9	
Moderate	8	12	
Low	9	24	
**Lymph node metastasis**			0.013
N0-1	17	14	
N2-4	11	31	
**Distant metastasis**			0.017
No	21	21	
Yes	7	24	
**TNM stage**			0.045
I–II	16	15	
III–IV	12	30	


**Table 4:** Univariate analysis of prognostic factors of hypopharyngeal cancer.

Variable	Hazard ratio	*p*-value
Age (≥60/<60)	0.95	0.328
Gender (male/female)	1.17	0.417
Tumor size (≥3 cm/<3 cm)	3.26	0.016
Differentiation (low/high-moderate)	2.26	0.023
Lymph node metastasis (N2-4/N0-1)	3.24	0.034
Distant metastasis (yes/no)	5.73	0.011
TNM stage (III–IV/I–II)	3.45	0.025
SNHG7 (low/high)	3.86	0.013


**Table 5:** Multivariate analysis of independent prognostic factors of hypopharyngeal cancer.

Variable	Hazard ratio	*p*-value
Tumor size	3.14	0.015
Differentiation	3.07	0.042
Lymph node metastasis	2.53	0.023
Distant metastasis	5.88	0.017
TNM stage	3.03	0.027
SNHG7 levels	3.26	0.024


### Metformin Sensitizes FaDu Cells to Taxol and Radiotherapy Through SNHG7

**FIGURE 5:**
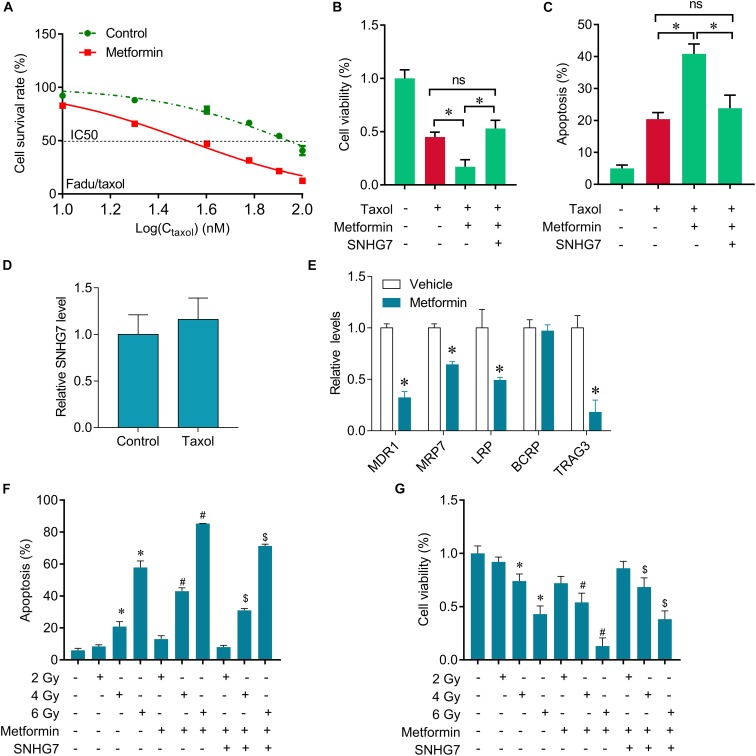
Metformin sensitizes FaDu cells to gefitinib through inhibiting SNHG7. **(A)** CCK-8 assay was used to determine the IC50 in FaDu/taxol cells (control vs. metformin: 79 vs. 31 mM). **(B)** CCK-8 assay was used to determine the change of viability after taxol, metformin and SNHG7 treatment. **(C)** Flow cytometry was used to measure cell apoptosis after taxol, metformin and SNHG7 treatment. **(D)** RT-qPCR analysis for SNHG7 in FaDu cells after treated with taxol. **(E)** RT-qPCR analysis for MDR1, MRP7, LRP, BCRP, and TRAG3 in FaDu cells after treated with or without metformin. **(F)** CCK-8 assay was used to determine the change of viability after irradiation, metformin, and SNHG7 treatment. **(G)** Flow cytometry was used to measure cell apoptosis after irradiation, metformin, and SNHG7 treatment. ^∗^*P* < 0.05 vs. control, ^#^*p* < 0.05 vs. irradiation, ^$^*p* < 0.05 vs. irradiation plus metformin; ns, no significance.

The expression of SNHG7 was significantly increased in the taxol resistant patients compared with the taxol sensitive patients, indicating that SNHG7 upregulation is involved in HPC chemotherapy resistance. FaDu/Taxol cells were treated with metformin. The IC50 was significantly decreased in cells treated with metformin compared with the cells treated with vehicle (Figure 5A). Additionally, we found that upregulation of SNHG7 largely attenuated the synergistic effects of metformin and taxol on cell proliferation inhibition and cell apoptosis induction (Figure 5B,C). We found that metformin could suppress drug resistant-related genes expression, including MRD1, MRP7, LRP and TRAG3, but not BCRP (Figure 5E), but Taxol treatment did not change the expression of SNHG7 (Figure 5D). Importantly, we further revealed that metformin treatment sensitized FaDu cells to irradiation, while upregulation of SNHG7 reversed the sensitization of metformin on radiotherapy evaluating by promotion of cell viability and reduction of cell apoptosis (Figure 5F,G). The results indicate that metformin enhances taxol and radiotherapy effects in HPC cells via targeting SNHG7.

## Discussion

In recent study, we observed that metformin could inhibit FaDu cell viability and significantly induce apoptosis by downregulating lncRNA SNHG7. Further investigations revealed that metformin decreased SNHG7 expression by activating SAHH activity and increasing DNMT1 expression.

Recent studies have shown that metformin has impacts on epigenomics by influencing the activity of epigenetic modifying enzymes such as AMPK and SAHH (Bridgeman et al., 2018). Activated AMPK phosphorylates many substrates and leads to epigenetic enzymes inhibition such as histone acetyltransferases and deacetylases, and DNA methyltransferases (DNMTs) (Ikhlas and Ahmad, 2017; Safe et al., 2018), which may contribute to protect against cancer, including HPC (Shan et al., 2016). LncRNAs are also influenced by metformin that confers anticancer activities. For example, metformin can disrupt the interaction between lncRNA MALAT1 and miR-142-3p to inhibit human cervical cancer cell growth (Xia et al., 2018). Metformin inhibited proliferation and glycolysis in bladder cancer cells through regulation of long non-coding RNA UCA1 (Li et al., 2017).

Here, we found that metformin decreased lncRNA SNHG7 expression by activating SAHH activity. SAHH is a potent feedback inhibitor of SAM-dependent methyltransferases. SAM methylases diverse cellular components, including DNA, RNA and proteins. We here show that metformin treatment activates SAHH, leading to increased DNMT1-mediated methylation of SNHG7. It was also found that activation of SAHH increased the expression of DNMT3a and DNMT3b in vascular smooth muscle cells HEK293 cells (Yideng et al., 2007; Zhou et al., 2015b). In recent study revealed that activation of SAHH induced DNMT1 expression in HPC cells. DNA methylation regulated by DNA methyltransferases (DNMT1, DNMT2, DNMT3a, and DNMT3b) is an important epigenetic mark that is typically associated with repressed genes. Genome-wide studies of DNA methylation in tumors have revealed that the promoters of some tumor-suppressor genes become hypermethylated and oncogenes are largely hypomethylated (Ehrlich, 2009; Wu and Zhang, 2014). Merry et al. demonstrated that deregulation of DNMT1-associated lncRNAs contributes to aberrant DNA methylation and gene expression during colon tumorigenesis (Merry et al., 2015). In several cancers, including colorectal cancer, prostate cancer, esophageal cancer, lung cancer and gastric cancer, lncRNA SNHG7 has been known as a potent oncogene by targeting GALNT1, Cyclin D1, FAIM2, p15 and p16 (She et al., 2016, 2018; Wang et al., 2017; Li et al., 2018; Qi et al., 2018; Xu et al., 2018). Recent study showed that knockdown of DNMT1 reversed metformin-mediated hypermethylation of SNHG7 promotor, while restoration of SNHG7 abolished metformin-induced inhibition of HPC cell growth.

In addition, we also observed that SNHG7 was significantly increased in HPC tissues compared with matched adjacent tissues, and was associated with advanced stage. Interestingly, SNHG7 levels were higher in taxol resistant patients than in taxol sensitive patients. The patients with high SNHG7 have lower overall survival than that of with low SNHG7. These results indicate that SNHG7 is an independent prognosis factor for HPC patients. Importantly, we demonstrated that metformin could sensitize FaDu cells to taxol and radiotherapy, a first line chemotherapy drug for cancers and the gold standard treatment for HPC (Zhang et al., 2014). Rescue of SNHG7 attenuated the synergistic effects of combination of metformin with taxol, which may be involved with the drug resistant-related genes, such as MRD1, MRP7, LRP, and TRAG3 (Hsiao et al., 2009; Sun et al., 2016). Our further study will demonstrate if this mechanism is typical of HPC in other cell lines and orthotopic implantation model.

## Conclusion

In conclusion, our recent study demonstrates that metformin inhibits FaDu cell proliferation by suppressing SNHG7 expression via SAHH-mediated DNA methylation. These findings indicate that combined metformin with paclitaxel would be a novel therapeutic strategy to overcome resistance and prevent recurrence in HPC.

## Author Contributions

PW and YT designed the study. XF, CX, and JZ analyzed and interpreted the patient data. PW, WW, and SZ performed cell biological experiments and *in vivo* experiments. XF, CX, and SZ performed qPCR, Western blot, RIP, and SAHH activity assay. All authors contributed to writing the manuscript. All authors read and approved the final manuscript.

## Conflict of Interest Statement

The authors declare that the research was conducted in the absence of any commercial or financial relationships that could be construed as a potential conflict of interest.
